# mSphere of Influence: a Sphere of Influence beyond the Bench Can Help Shape the Future of U.S. Research

**DOI:** 10.1128/mSphere.00318-19

**Published:** 2019-07-03

**Authors:** Megan E. Spurgeon

**Affiliations:** a McArdle Laboratory for Cancer Research, Department of Oncology, University of Wisconsin—Madison School of Medicine and Public Health, Madison, Wisconsin, USA

**Keywords:** biomedical research, career

## Abstract

Megan E. Spurgeon works in the field of viral oncology. In this mSphere of Influence article, she reflects on how the paper “Rescuing US biomedical research from its systemic flaws” by Bruce Alberts, Marc W. Kirschner, Shirley Tilghman, and Harold Varmus (Proc Natl Acad Sci U S A 111:5773–5777, 2014, https://doi.org/10.1073/pnas.1404402111) made an impact on her by influencing her research and career outlook. The way researchers think about their science is shaped by a variety of factors, and these often include research articles that influence their technical or experimental approaches and interpretations. However, it is rare that an article influences the broader career and research perspectives of scientists by compelling them to look beyond the bench. The article highlighted in this commentary prompted the realization that a sphere of influence beyond our routine scientific focus can create a scientific community that is better equipped to address the systemic threats facing the larger research ecosystem to which we all belong.

## Abstract (precis)

Megan E. Spurgeon works in the field of viral oncology. In this mSphere of Influence article, she reflects on how the paper “Rescuing US biomedical research from its systemic flaws” by Bruce Alberts, Marc W. Kirschner, Shirley Tilghman, and Harold Varmus (Proc Natl Acad Sci U S A 111:5773–5777, 2014, https://doi.org/10.1073/pnas.1404402111) made an impact on her by influencing her research and career outlook.

## COMMENTARY

In research, we quickly become absorbed in our own realm of study. Throughout our training and continuing into our research careers, we develop an increasingly concentrated and specialized niche. Gradually, we become fully engrossed in this area, and we adopt a routine in which we almost exclusively focus on our own experiments, projects, laboratory, and related literature. It was during such a period of scientific self-centeredness that I noticed some chatter on social media about an article making waves in the scientific community. The article “Rescuing US Biomedical Research from Its Systemic Flaws” by Bruce Alberts, Marc Kirschner, Shirley Tilghman, and Harold Varmus was published in the *Proceedings of the National Academy of Sciences* in 2014 (1). In their article, these prominent scientists outline flaws that have become inherent in the U.S. system of biomedical research and threaten its long-term stability and success. Alone, each of these threats to research elicited reflection and concern. Collectively, however, the threats outlined in the whole article had a considerable influence on my perspective, outlook, and approach to my research and career. It was in reading this article that I realized the need to modify our insular routines in order to become equipped to understand, critique, and improve the research enterprise within which we are all functioning.

The primary theme of the article is unapologetically blunt: the U.S. biomedical research enterprise is on an unsustainable path. The authors approach this delicate topic using a logical and effective strategy. They first explain the reasons why and how the current path is structurally unsound. Their view of what caused this dilemma, including an unreasonable expectation of continued expansion of the research enterprise, the rising cost of research, and a large imbalance between supply and demand in both funding and the scientific workforce, is clearly presented. The authors identify the main systemic flaws threatening our research system. Using what I found to be a particularly powerful and compelling strategy, they not only present the main idea of each flaw, they also methodically expose how they result in a cascade of negative and interwoven downstream effects. For instance, they reveal how hypercompetition for research funds and positions has resulted in intense pressure to publish manuscripts that can be coupled with reduced quality and veracity of research. These effects, in turn, fuel other flaws in the system, such as the overwhelming strains on scientists’ time. Increasing incentives for academic research institutions to use grants to support faculty and staff salary in lieu of institutional support has created large ranks of employees that exist in unstable “soft money” positions. These and other effects culminate in what the authors view as the most problematic potential outcome of this dilemma, which is that young scientists will broadly reject academic research as a career prospect given its unappealing and discouraging environment. In diplomatic yet frank terms, the authors conclude by outlining strategies to address these systemic flaws.

I am not highlighting this article as a way to openly endorse its policy prescriptions, although there are certainly proposed solutions I support. Instead, I sought to highlight this article as part of my mSphere of Influence, because it served as a wake-up call that, as scientists, we all exist and function within a larger ecosystem, and with this comes a collective responsibility to contribute to its long-term sustainability. It forced me to view both my research and career within a larger context. I was no longer content to exist idly within the system, but instead I wanted to educate myself so I could actively participate in the conversations stimulated by this article about exploring new paradigms. At the time the article was published, its effect on my thinking was likely amplified by my being a young researcher transitioning from a postdoctoral position to a staff scientist position at a public academic research institution, all key demographics poised to reap the consequences of inaction. I therefore began my position as a staff scientist with a more critical interest in the history and policies of the research system in which I was trained and was now choosing to continue my career. In large part due to this article, my career emphasis has evolved. While the majority of my daily focus remains bench research, I have purposefully tried to resist the habit of being self-absorbed in my own science. I have become more interested and involved in academic staff and policy issues at the University of Wisconsin—Madison in a variety of ways: I helped cofound the University of Wisconsin Scientist Network to encourage a discussion on issues important to staff scientists, I have become more involved in shared governance and now serve on my school’s Committee on Academic Staff Issues, and I have served on committees considering the introduction of a Research Professor track at the university as a way to provide a viable, attractive career option for outstanding and productive staff scientists.

A national conversation about the U.S. biomedical enterprise was sparked by publication of this article (1), and discussion continues to flourish. There was a range of initial reactions to the report and its proposals (2–4), and there have been several efforts to promote continued dialogue. The four authors and a steering committee of scientists created a website (www.rescuingbiomedicalresearch.org) for discussion, suggestions, and updates. The authors also convened a follow-up workshop with established scientists to discuss its contents and proposals (5). Junior scientists and postdoctoral researchers have also joined the conversation, and the Future of Research organization (www.futureofresearch.org) continues to serve as a platform for the perspectives of young scientists (6, 7). Here at the University of Wisconsin—Madison, we held a series of campus-wide workshop discussions on the topic (8). Moving forward, addressing an issue of this magnitude and formulating realistic and effective solutions will require that scientists at all levels, and especially young scientists, take the initiative to look beyond the bench and get involved. For me, reading this article was a good start.
